# Signaling at the Root Surface: The Role of Cutin Monomers in Mycorrhization

**DOI:** 10.1093/mp/sst090

**Published:** 2013-08-10

**Authors:** Jeremy D. Murray, Donna R. Cousins, Kirsty J. Jackson, Chengwu Liu

**Affiliations:** Cell and Developmental Biology, John Innes Centre, Norwich NR4 7UH, UK

Most vascular plants interact with arbuscular mycorrhizal fungi (AMF) and are thereby provided significant advantages in nutrient acquisition, especially phosphate. The widespread retention of this symbiosis in vascular plants is testament to its importance and ancient origins. This highlight focuses on the reports of two of the first genes identified through forward genetic screens for AMF symbiotic mutants: *Required for Abuscular Mycorrhization 1* (*RAM1*) and *RAM2*, which respectively encode a GRAS transcription factor and a glycerol-3-phosphate acyl-transferase (GPAT) that are required for the formation of fungal entry structures (hyphopodia) on the root surface. This provides the first insights into a mycorrhization specific signaling pathway and reveals cutin monomers as a critical component of signaling in the mycorrhizal symbiosis. We discuss the proposed links between these genes, and the role of cutin and its precursors in interactions with AMF, and oomycete and fungal pathogens.

Root colonization by AMF involves the detection of compounds found in host root exudates such as strigolactones. Strigolactones are carotenoid-derived terpenoids that serve as a rhizosphere signal to activate responses in AMF, including hyphal branching, and thereby promote mycorrhizal colonization of the root surface. This involves the fungus making contact with the plant epidermal cell wall and forming a hyphopodium which is a lobed hyphal contact point with the root that serves as the entry point of the fungus into the epidermis. The hyphopodium is a specialized structure similar to, but distinct from, the pathogenic appressorium. The fungus then passes through an epidermal cell and colonizes the root cortex by extensive intercellular hyphal growth and the formation of terminal intracellular structures called arbuscules. The arbuscule, which serves as the nutrient exchange interface in the symbiosis, is highly branched and is surrounded by plant plasma membrane.

It has been known for some time that the evolutionary history of the mycorrhizal symbioses is closely intertwined with that of a second root endosymbiosis called nodulation. The signaling required for the initiation of nodulation in legumes involves several genes that are also essential for mycorrhization (Kistner et al., 2005). This common set of genes is usually referred to as the common signaling pathway. For clarity, throughout this manuscript, common signally pathway genes identified in model legumes will be given as *Medicago truncatula*/*Lotus japonicus*. These shared components comprise a signaling circuit, which includes the ion channel *DMI1* (*Does not Make Infections*)*/POLLUX*, *DMI2/SYMRK* (*SYMbiosis Receptor like Kinase*) and the calcium calmodulin kinase *DMI3/CCaMK* (Catoira et al., 2000) and its substrate *IPD3* (*Interacting Protein of DMI3*)/*Cyclops.* Downstream of this shared signaling pathway lie the genes regulating the transcriptional outputs, including the GRAS transcription factors NSP1 and NSP2, which interact to regulate expression of nodulation genes (Hirsch et al., 2009).

## MYCORRHIZATION INVOLVES RAM1 REGULATION OF *RAM2* EXPRESSION

**Figure 1.**
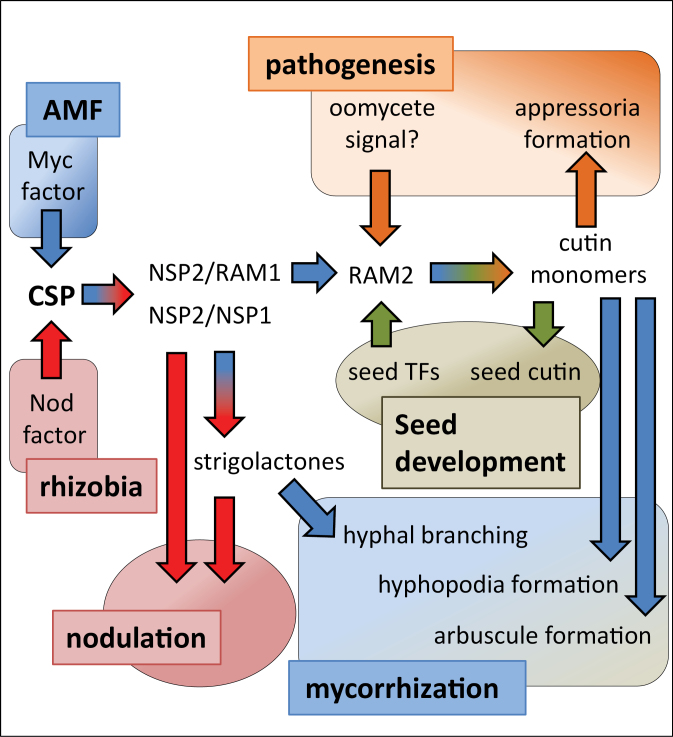
RAM1 and RAM2 Have Roles in Cutin Monomer Biosynthesis. Myc or Nod factors activate the common symbiosis pathway (CSP) to activate the GRAS transcription factors NSP1, NSP2, and RAM1. *NSP1* and *NSP2* have been shown to be involved in strigolactone biosynthesis (Liu et al., 2011). In roots, cutin monomers produced by RAM2 promote appressorium formation by *P. palmivora* and may play an analogous role in hyphopodia formation by AMF. In the latter case, *RAM2* expression is dependent on *RAM1*. In seeds, *RAM2* expression may be under developmental control and the cutin monomers produced may get incorporated into cutin.

Gobbato et al. (2012) and Wang et al. (2012) show that mutations in *RAM1* or *RAM2* lead to a drastic decrease in both mycorrhizal colonization and arbuscule formation but did not affect nodulation. It was further shown that reduced colonization in *ram1* and *ram2* mutants is accompanied by impaired hyphopodia formation. Overexpression of *RAM2* led to an increase in levels of α,ω-dicarboxylic acids and ω-hydroxy fatty acids—a profile equivalent to that produced by overexpression of an *Arabidopsis* GPAT involved in cutin biosynthesis. Both *ram1* and *ram2* were found to induce normal AMF branching responses, suggesting that their strigolactone production was normal. In addition to its role in mycorrhization, *RAM2* was shown to be required for appressoria formation by an oomycete pathogen. A link between RAM1 and RAM2 was revealed by Gobbato et al. (2012), who showed that the induction of *RAM2* by AMF was dependent on *RAM1*. This connection was further strengthened the demonstration of a direct *in vivo* interaction between RAM1 and the *RAM2* promoter using chromatin immunoprecipitation. The authors further showed that RAM1 is capable of interacting with NSP2 in a yeast two-hybrid assay and by using bimolecular fluorescence complementation. This suggests that RAM1 may serve an analogous role in mycorrhization to NSP1 in nodulation and, as suggested by Gobbato et al. (2012), that perhaps RAM1 and NSP1 could compete for binding with NSP2, thereby providing a mechanism to regulate symbiosis-specific transcription (Figure 1). Intriguingly, NSP1 and NSP2 have recently been shown to be involved in the induction of *DWARF27*, a gene involved in strigolactone production (Liu et al., 2011). Therefore, it seems clear that, even though RAM1 and NSP1 both interact with NSP2, they regulate the expression of different target genes: NSP1 is needed for strigolactone production and RAM1 controls expression of *RAM2*.

## THE REGULATION OF RAM2 EXPRESSION BY RAM1 IS LIMITED TO MYCORRHIZA

Differences in the *ram1* and *ram2* mutant phenotypes suggest that the regulation of *RAM2* expression is context-dependent. One difference between the *ram1* and *ram2* mutants was that *ram2* had dark pigmented seeds that were permeable to water-solubilized dye, similarly to the *Arabidopsis gpat5* mutant, while *ram1* seeds were normal. This strongly suggests that RAM2 is still functional in the *ram1* mutant seeds, so therefore its expression must be dependent on another transcription factor in this organ. This also shows an effect for RAM2 on the physical characteristics of the seed (water permeability), which is presumably a consequence of reduced cutin formation. Another context where *RAM2* expression appears to be independent of RAM1 is during interactions with the oomycete pathogen *Phytophthora palmivora. P. palmivora* fails to form appressoria on *ram2* whereas *ram1* supports normal infection demonstrating that cutin monomer signaling is intact in *ram1*. Finally, the reduced hyphopodium formation on *ram1* and *ram2* contrasts markedly with the *dmi1/pollux*, *dmi2/symrk*, and *dmi3/ccamk* signaling pathway mutants which have normal or increased hyphopodium formation (Kistner et al., 2005). This seems to be inconsistent with the induction of *RAM2* expression being dependent on DMI2/SYMRK and DMI3/CCaMK (Wang et al., 2012) but could be explained by common symbiosis pathway-independent expression of *RAM2* in the epidermis. In this scenario, the common symbiosis pathway regulation of *RAM1* and *RAM2* would be restricted to the cortical cells to control arbuscule formation. Expression studies to localize *RAM2* transcripts would help clarify this issue. In summary, it would appear that *RAM2* expression is under complex regulation which is needed to mediate its roles in mycorrhization, seed development, and plant–pathogen responses (Figure 1).

## CUTIN MONOMERS AS A PLANT-TO-MICROBE SIGNAL

Cutin is a product of esterification of cutin monomers into polyester compounds. This waxy and water-repellent substance is found mostly in the aerial tissues of plants, where it serves to prevent moisture loss. Cutin is enriched in the cuticle layer which is sandwiched between an outer layer of epicuticular waxes and an inner layer of cutin, waxes, and carbohydrates anchored to the cell wall. In fungal pathogenesis, both cutin and its precursors have been shown to play a role. Cutin provides both a hydrophobic surface which is needed for the early stages of the interaction and a substrate for fungal cutinases, the latter serving to generate local cutin monomer signals needed for host recognition. In the Wang et al. (2012) study, a role for cutin monomers in signaling during oomycete interactions was indicated by the inability of *P. palmivora* zoospores to form appressoria on *ram2* roots despite successful germination. Addition of C16:0 cutin monomers rescued the root infectivity of *P. palmivora* and also significantly enhanced appressoria formation on a polypropylene surface. The addition of cutin monomers alone was not sufficient to induce zoospore germination (S. Schornack, personal communication). These findings appear to mirror studies of the fungal pathogen *Ustilago maydis.* It was shown that, for *U. maydis*, cutin monomers were able to induce filamentation and to promote appressoria development provided that a hydrophobic surface was present (cutin monomers alone were not effective; Mendoza-Mendoza et al., 2009). Moreover, they showed that these responses were dependent on the *U. maydis* MAP kinase Kpp2. The source of the cutin monomers in pathogenic interactions sometimes comes from the degradation of surface cutin. A *Magnaporthe grisea* cutinase mutant has been shown to have reduced pathogenicity and notably it can have its virulence rescued by application of cutin monomers (Skamnioti and Gurr, 2007). The Wang et al. (2012) study supports the idea that AMF also perceive the cutin monomers as a signal, but more experimentation is needed to show that mycorrhizal fungi can respond to these compounds in absence of the host.

In general, roots do not contain cutin, but instead contain the related compound suberin. The use of cutin compounds that are otherwise absent in the root may provide a specific cue for the fungus—one that apparently some pathogens can exploit. From an evolutionary standpoint, it is interesting to note that early land plants lacking roots had interactions resembling mycorrhizas in their rhizomes, which are modified stems (Brundrett, 2002). These early associations may explain why cutin monomers, which are normally found in aerial tissues, have taken on a role in a root symbiosis.

## FUNDING

This work was supported by BBSRC grants BB/J004553/1 and CA403A13B.
